# Multiple lumbar punctures aiming to relieve headache results in iatrogenic spinal hematoma: a case report

**DOI:** 10.1186/s13256-022-03687-y

**Published:** 2022-12-14

**Authors:** Hannah S. Lyons, Satheesh Ramalingam, James L. Mitchell, Andreas Yiangou, Mark Thaller, Alexandra J. Sinclair, Susan P. Mollan

**Affiliations:** 1 Translational Brain Science, Institute of Metabolism and Systems Research, College of Medical and Dental Sciences, University of Birmingham, Birmingham, B15 2TT UK; 2 Department of Neurology, University Hospitals Birmingham NHS Foundation Trust, Queen Elizabeth Hospital, Birmingham, B15 2WB UK; 3 Centre for Endocrinology, Diabetes and Metabolism, Birmingham Health Partners, Birmingham, B15 2TH UK; 4 Department of Interventional Radiology, University Hospitals Birmingham NHS Foundation Trust, Queen Elizabeth Hospital, Birmingham, B15 2WB UK; 5 Birmingham Neuro-Ophthalmology, Queen Elizabeth Hospital, University Hospitals Birmingham NHS Foundation Trust, Birmingham, UK

**Keywords:** Spinal hematoma, Lumbar puncture, Complications, Cauda equina syndrome, Idiopathic intracranial hypertension, Headache, Case report

## Abstract

**Background:**

Multiple lumbar punctures have historically been a strategy to relieve headaches associated with idiopathic intracranial hypertension despite limited clinical evidence of long-term efficacy. Lumbar puncture is typically a straightforward procedure with minimal complications reported, however, serious complications can occur. Lumbar-puncture-related spinal hematomas are rare but can lead to irreversible paralysis.

**Case presentation:**

We report a case of a 28-year-old Caucasian woman who was treated with multiple lumbar punctures to manage headache, thought to be attributed to idiopathic intracranial hypertension. The patient developed a lumbosacral epidural hematoma following a lumbar puncture, which led to incomplete cauda equina syndrome. Multiple lumbar punctures had been the long-term management for the patient’s chronic headaches associated with her diagnosis of idiopathic intracranial hypertension. She had no risks of an underlying coagulopathy. Following a lumbar puncture, she re-presented with lower back pain and bilateral paresthesia. Over the subsequent 48 hours, this progressed to urinary incontinence and saddle paresthesia. Imaging revealed an epidural hematoma, which was conservatively managed. She continued to report saddle paresthesia and urinary incontinence 7 months following the lumbar puncture. Between 1974 to 2022, our literature search found 41 case reports detailing lumbar-puncture-related spinal hematomas. It is an established but rare complication of lumbar puncture and there are limited studies looking at the incidence of its occurrence. Whilst coagulopathy has been found to be a risk factor, it is unclear if the gauge of the needle is relevant. Case evidence suggests there may be no significant difference in outcomes between surgical and conservative management of spinal hematomas. This case highlights that lumbar punctures can be invasive, with potentially serious complications. A lumbar puncture should therefore only be performed when clinically justified.

**Conclusions:**

This case highlights a rare complication of lumbar puncture and emphasizes the importance of a risk–benefit discussion for each procedure. Spinal hematoma following lumbar puncture is a rare complication but with potentially devastating consequences. Within the setting of idiopathic intracranial hypertension, the evidence base for the long-term benefit of headache relief by repeat lumbar puncture is low.

## Case report

### Background

Lumbar puncture (LP) is typically performed to aid diagnosis of the underlying etiology. LPs have been used to therapeutically decrease the volume of cerebrospinal fluid (CSF) and intracranial pressure; however, reduction in CSF is transient as CSF is rapidly replaced at a rate of 0.3 to 0.4 ml per minute [1]. Historically, in disorders of elevated increased intracranial pressure (ICP), multiple LPs were performed despite a lack of evidence base for efficacy [2]. Multiple LPs are no longer advised for the treatment of idiopathic intracranial hypertension (IIH) and have been shown to lack efficacy to treat headache in IIH long term [2, 3].

Typically, LP is a relatively straightforward procedure with minimal complications. Common complications include back pain, minor bleeding at the skin site, and a post-dural puncture headache (< 70%) [4, 5]. Rare complications include infection, damage to local structures such as nerves, and spinal hematoma [6]. The location of a spinal hematoma can be epidural, subdural, and subarachnoid. They can result in a range of features from mild pain with subsequent resolution without complications, to exerting a mass effect on the nerve roots or spinal cord, causing paralysis and rarely death [7]. Early diagnosis is important to facilitate timely intervention to prevent irreversible paralysis. Herein we report a case of a patient who was treated with multiple LPs to manage headache attributed to IIH who developed an epidural hematoma. This resulted in incomplete cauda equina syndrome with permanent urinary incontinence.

### Case presentation

A 28-year-old Caucasian woman, with a background of meningitis as a child, subsequently developed raised intracranial pressure and had a ventriculoperitoneal (VP) shunt inserted at 7 years old. It is unclear from the historical notes as to whether this was primary IIH or secondary intracranial hypertension (given the prior medical history of meningitis). Over the subsequent years, she suffered with headaches in the absence of papilledema and underwent multiple VP shunt revisions. She reported severe debilitating chronic headaches, which led to frequent visits to the emergency department. During these admissions, LP was performed on more than 15 occasions. She reported that this gave her temporary relief from her headaches for a week or so at a time. Her medication history includes amitriptyline, levothyroxine, and sertraline. She was not on any formal headache medicines. She took simple analgesics, using codeine and paracetamol as required.

At the present day she visited the emergency department with blurring of her vision, right-sided weakness, and exacerbation of headache. Shunt dysfunction was questioned and she underwent magnetic resonance imaging (MRI) brain, which showed stable appearances of her ventricles. An LP was then performed on the ward in the left lateral decubitus position using a pink Quincke needle (18G with cutting bevel). The LP was a traumatic tap evidenced by blood-stained fluid. An opening pressure of 15 cm CSF was recorded. CSF constituents were normal except for an elevated red cell count. Her clotting and biochemical bloods were normal and she was discharged.

**Fig. 1:**
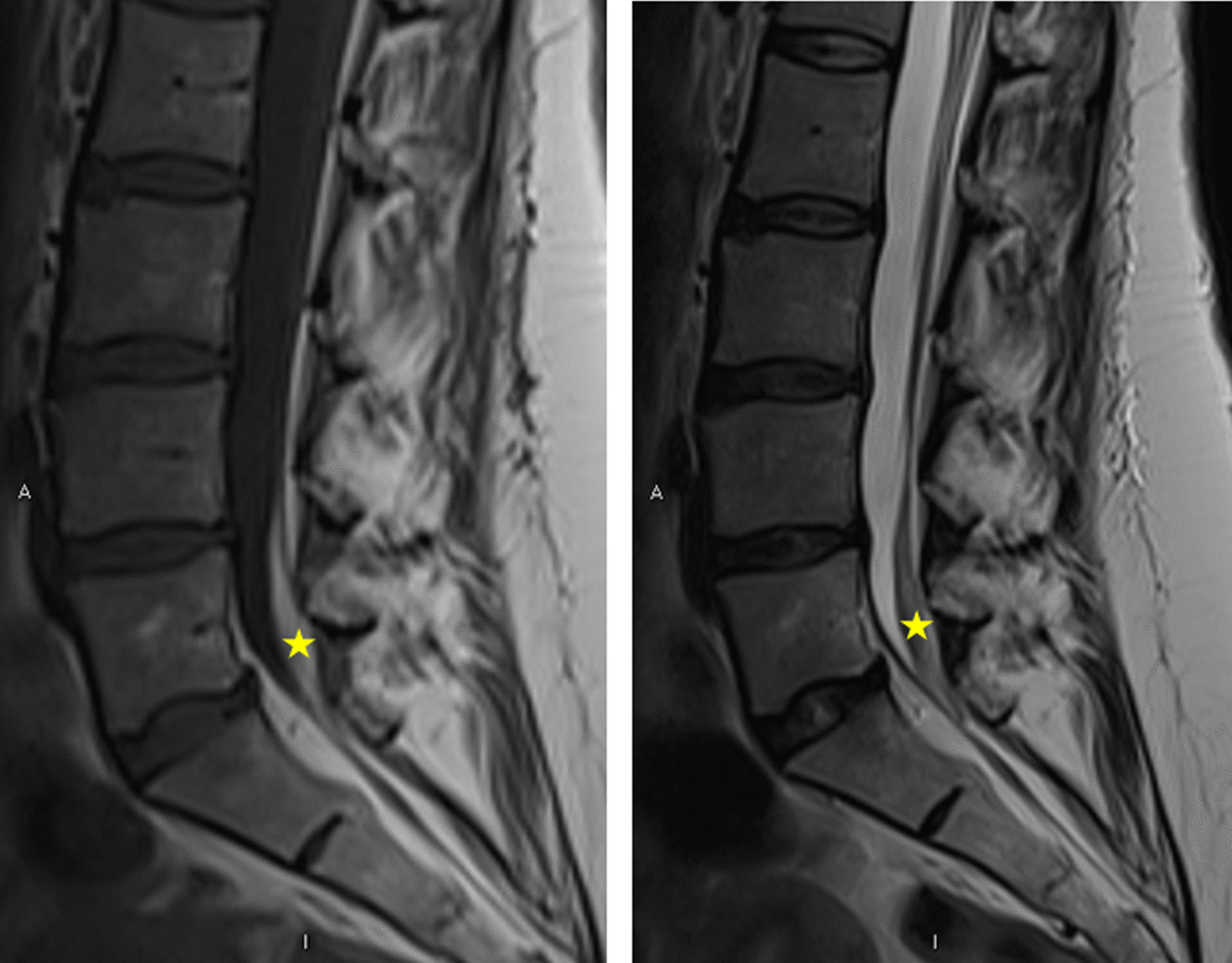
Initial images: Sagittal magnetic resonance imaging lumbar spine T1-weighted (left) and T2-weighted (right). There is a small volume T1 hyperintense intradural blood clot (asterisk) intermeshed with cauda equina nerve roots in posterior aspect of the thecal sac at L5 vertebral level. On the T2 weighted sequence, the clot appears relatively hypointense to the cerebrospinal fluid

**Fig. 2:**
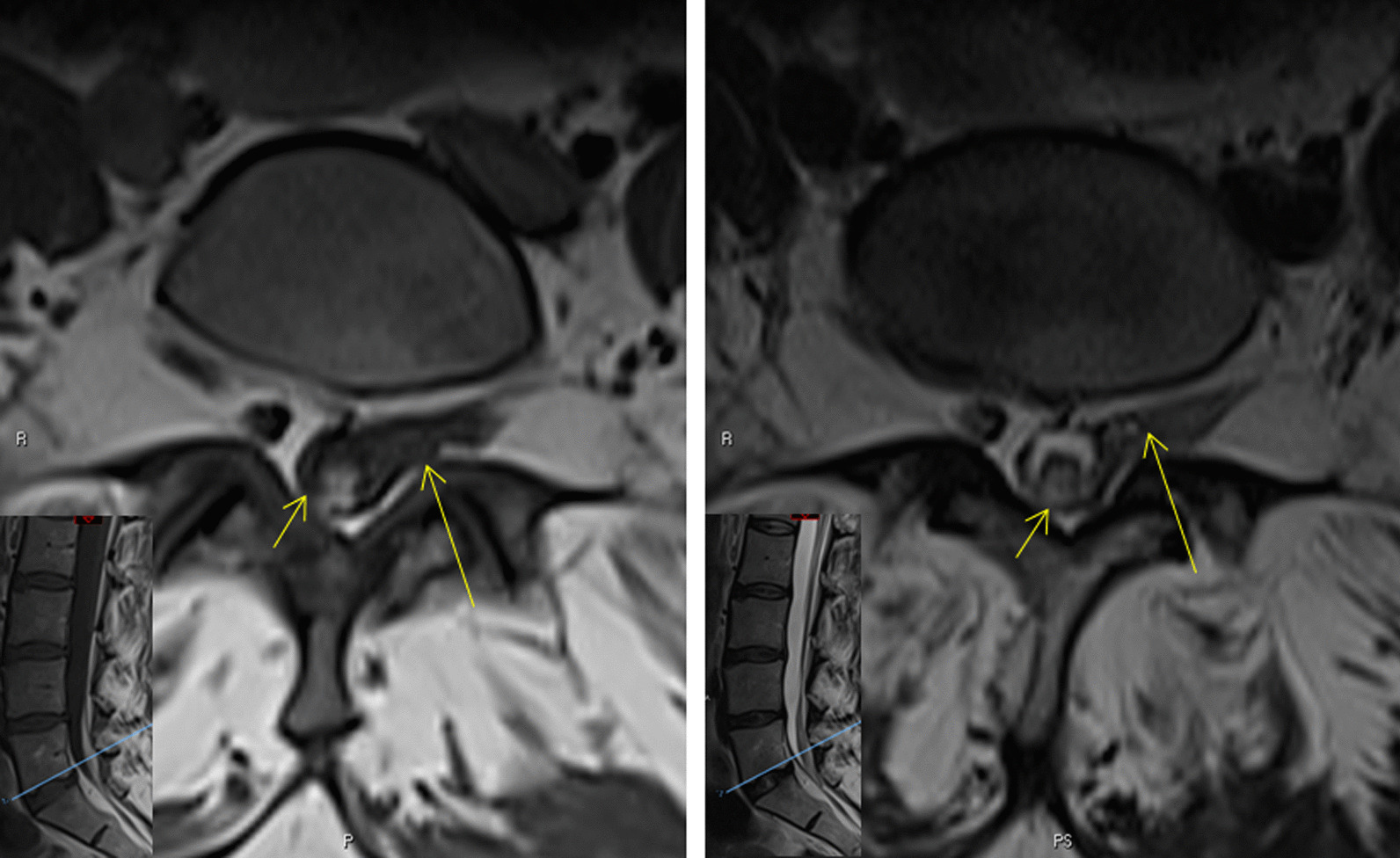
Initial images: Axial magnetic resonance imaging lumbar spine T1-weighted (left) and T2-weighted (right). On the axial images, in addition to the intrathecal blood clot (short arrow), there is perineural inflammatory stranding (long arrow) around the exiting left S1 nerve root fascicles

Five days after the LP she was re-admitted with new bilateral shooting leg pains and lower back pain. She was discharged with the plan to have an urgent MRI spine as an outpatient. However, the following day she returned with new and unprovoked episodes of urinary incontinence, lower back pain, and saddle paresthesia. She underwent an urgent MRI spine which revealed a small 4 cm epidural lumbosacral hematoma at L1/S1, as shown in Figs. 1 and 2.

**Fig. 3:**
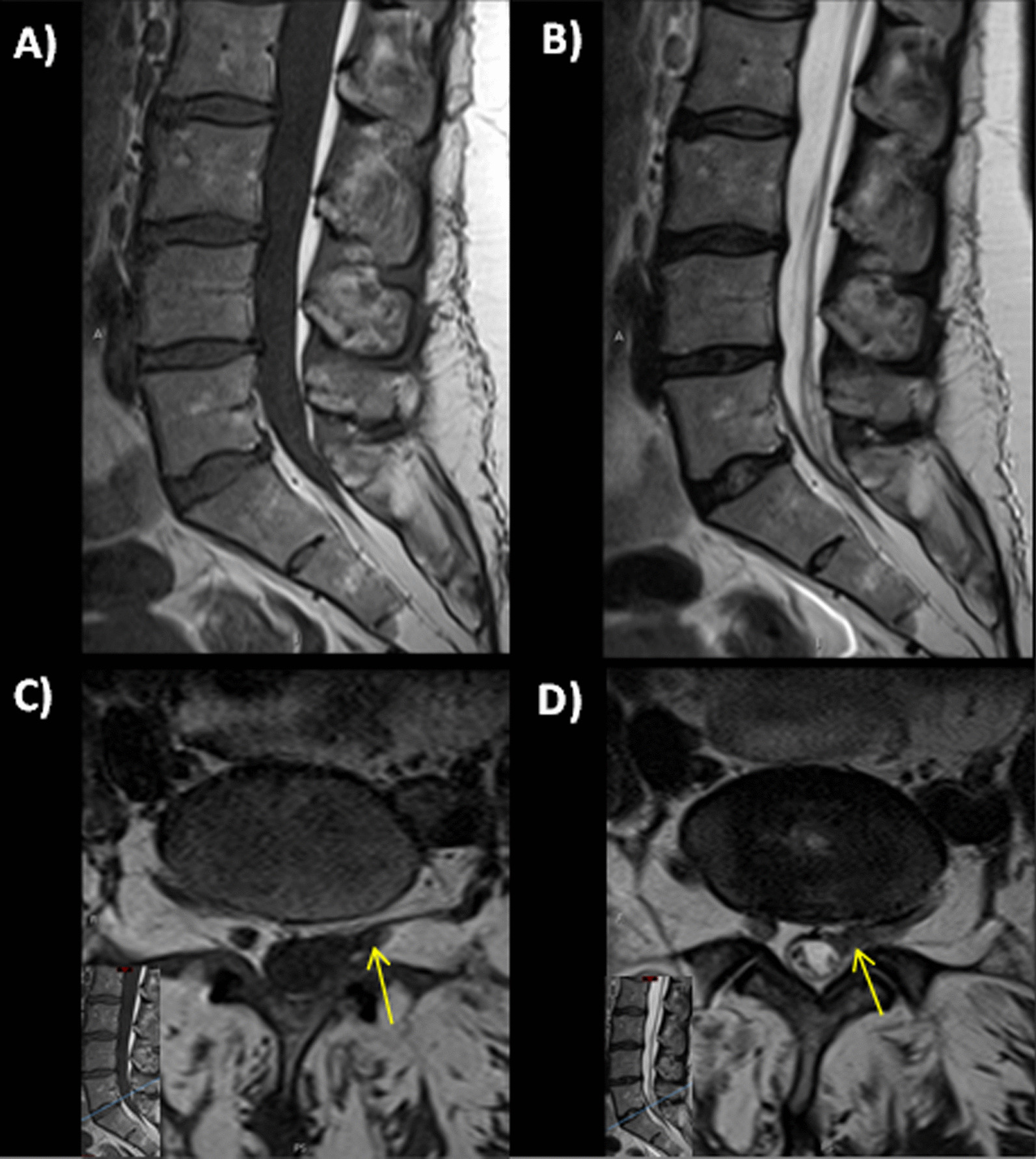
Magnetic resonance imaging lumbar spine images 4 months later; **A** Sagittal T1-weighted; **B** Sagittal T2-weighted; **C** Axial T1-weighted; **D** Axial T2-weighted. Follow-up study performed 4 months later shows clearance of the intradural blood clot with minor residual clumping of the cauda equina nerve roots within the thecal sac (arrow)

She was diagnosed with incomplete cauda equina syndrome secondary to a hematoma caused by the LP performed a week earlier. On advice from the neurosurgical team she was managed conservatively. She was prescribed senna and lactulose to prevent constipation, and she was monitored as an outpatient. A repeat MRI spine 4 months later showed a small residual spinal hematoma with substantial reduction in size, as shown in Fig. 3. There was minor abutment on the left S1 nerve root.

She was referred to the specialist IIH clinic and seen 7 months later, where it was deduced that her headaches met the International Headache Society Criteria of chronic migraine with aura, and were not associated with raised intracranial pressure. She reported permanent deficit of sensation in the saddle region and right leg with ongoing urinary incontinence.

## Discussion

Cauda equina syndrome secondary to post-LP epidural hematoma is rare but can have devastating implications, including paraparesis and incontinence, as evidenced by this case. Conducting serial LPs to manage headache in IIH without clear evidence is not recommended [2]. This is partly due to the rapid rate at which CSF is secreted from the choroid plexus; thereby, the CSF removed in a “therapeutic-tap” would be quickly replaced [5], but also the risk of back pain post-LP (reported as 17% by Duits *et al*.) [8]. We appreciate that headache intensity can improve in the short term, with a Danish study showing 72% of patients with IIH having an improvement in headache following CSF withdrawal (10–15 minutes) and a UK clinical study showing improvement in 71% IIH patients post-LP (7 days) [3, 9]. The UK study also found that 64% of their IIH participants experienced a headache exacerbation over the following week post-LP [3]. Interestingly, Yri *et al*. also found nearly a quarter of the control participants had improvement of headaches following LP. Headache improvement post-LP has only been prospectively looked at in the short term and improvement was minimal (mean reduction one point on numerical rating scale) [3]. In our case, given that the opening pressure was 15 cm CSF, it was deduced that her headaches were not currently caused by ongoing raised intracranial pressure. At the follow-up appointment she was started on acute and prophylactic treatment for chronic migraine.

The side effects of LP are well documented, as is the negative patient experience of LP in those with IIH [10]. They include minor complications such as headache (40–70%) and back pain (20–40%) [4, 11]. Serious complications include severe back or lumbosacral radicular pain (10%), alongside paraparesis, infection, and cerebral herniation (< 0.01%) [11, 12]. Rarely, spinal hematoma can occur, but the risk of spinal hematoma is challenging to quantify as there is minimal literature on the complication. A Danish cohort study described the risk of spinal hematoma following lumbar puncture was 0.20% among patients without coagulopathy and 0.23% among those with coagulopathy [13]. Following epidural or spinal anesthesia, the reported incidence ranges from 1:1341 to 1:200,000 [7, 14, 15]. If this rare complication does occur, the impact on the patient should be considered, including muscle weakness or paralysis, difficulty walking, and incontinence.

To minimize the risk of a spinal hematoma, it is important that patients have their blood taken pre-procedure in order to ensure no coagulopathy [16]. Approximately 40% of patients who develop LP-related spinal hematoma have an underlying coagulopathy or iatrogenic administration of an anticoagulation prior to LP [7]. The use of anticoagulants is known to increase the risk of developing a spinal hematoma [11]. Brown *et al*. evaluated 35 LP-related spinal hematoma cases from 1974 to 2014. He found that only 14.3% of those without coagulopathy had poor outcomes regardless of intervention, compared with 28.6% with preexisting coagulopathy at 12 months. He noted no significant differences in sex, with a mean age of 48 years old (range 17 months to 83 years) [7].

Using a midline technique to approach the LP minimizes the risk of trauma to the artery and vein of Adamkiewicz that are implicated in spinal bleeding. There has been no meaningful association between spinal needle diameter and type to the development of a spinal hematoma [7, 17]. Some reports have stated that a traumatic LP increases the risk of developing an extradural spinal hematoma in patients with or without anticoagulation [11]. Brown *et al*. found that of the 35 LP-related hematoma cases, 34.4% were traumatic and 20% were atraumatic. This data also included unsuccessful attempts [7]. At present in certain health care settings, atraumatic needles are more expensive than standard needles such as Quincke [18].

It is essential to recognize a spinal hematoma as a complication of LP to expedite neurological examination and subsequent imaging. The documented evidence of a bloody tap in this case could have alerted the emergency department doctors to this complication. An MRI spine enables prompt diagnosis of acute epidural hematomas [19]. Sklar *et al*. determined the “typical” MRI features of 17 patients with acute spinal epidural hematomas. They reported the following features: (a) variable signal intensity, (b) capping of epidural fat, (c) direct continuity with adjacent osseous structures, (d) compression of epidural fat, subarachnoid sac, and spinal cord, and (e) location usually posterolateral in the spinal cord [19].

The management of an epidural hematoma is similar to that of a hematoma resulting from other causes. Despite spontaneous remission of some hematomas, others may require early surgical decompression [20]. There is a lack of research to support surgical versus conservative treatment for spinal hematomas post-LP. There are different opinions on whether time-to-treat alters the outcome. Some have found statistically significant improvements in outcomes for those who receive early surgical intervention [21]; whereas others have not found statistical improvement in outcomes at 12 months [7, 22]. Conservative treatment may be appropriate for those who have mild symptoms and show early signs of recovery. Some cases use dexamethasone in their management paradigm [17]. Overall, the most important prognostic factors are time from LP to diagnosis, time from diagnosis to intervention, and extent of neurological symptoms at presentation [23].

Since Brown *et al*.’s study [7], six further case studies have reported an iatrogenic spinal hematoma following an LP as presented in Table 1 between January 2014 and January 2022.

**Table 1:** Reported case studies of iatrogenic spinal hematoma following a lumbar puncture, January 2014–January 2022

Case study	Procedure	Clotting defect	Time to presentation	Pain	Red cell count in CSF (μL)	Hematoma	Management	Outcome
Bi *et al*., 2021 [22]	Epidural anesthesia	None	Unknown	Unknown	Unknown	SDH	Conservative	Fully recovered
Sawaya *et al*., 2018 [13]	Epidural anesthesia	Normal clotting bloods but on LMWH with AKI	1 hour	Headache	Unknown	IDH + EDH	Conservative	Unknown
Sawaya *et al*., 2018 [13]	LP	Normal blood but had myelodysplasia	Few hours	Lower back pain and bilateral thighs	4	IDH	Conservative	Death
Park *et al*., 2017 [23]	LP	None	Several minutes	Unknown	Unknown	SAH	Surgery	Fully recovered
Kothari *et al*., 2016 [21]	LP	Yes, mildly decreased fibrinogen only	Several hours	Bilateral legs	Unknown	SAH + SDH	Surgery	Unknown
Avecillas-Chasin *et al*., 2016 [24]	Spinal anesthesia	Unknown	3 days	Lower back and legs	Unknown	SAH + SDH	Surgery	Mild paraparesis

*CSF*  cerebrospinal fluid, *LP*  lumbar puncture, *SAH*  subarachnoid hematoma, *SDH*  subdural hematoma, *IDH*  intradural hematoma, *EDH*  epidural hematoma, *LMWH*  low molecular weight heparin, *AKI* acute kidney injury

Of these six patient cases, only one had confirmed derangement of clotting factors (mild) [24], whereas the two cases in Sawaya *et al*. had risk factors—myelodysplasia and the use of LMWH with an AKI [16]. A total of 50% were managed conservatively and 50% were managed surgically. The sample sizes are too small to make any other inferences.

In the case presented, the woman was referred to the IIH clinic for management of her headache rather than habitual visits to the emergency room for LP. Spinal hematoma remains a rare complication of LP, with controversy in how to best manage it to prevent long-term morbidity. The IIH consensus guidelines recommend that serial LPs are not indicated for headaches, as it only provides short-term relief with no evidence of long-term reduction in headache burden [2].

There are limited studies on spinal hematoma post-lumbar puncture and the Danish cohort study may reflect bias due to physicians selecting relatively low-risk patients for lumbar puncture[13]. Additionally, there are no clinical trials that look at the efficacy of LP in treating IIH, or indeed the effect of size of LP on patients with IIH. This may be a focus of future clinical investigation.

## Conclusions

This is a rare case of a spinal hematoma following a LP. The key learning points of this case are that there should be a risk–benefit discussion at the time of taking consent that includes a discussion of rare complications of the procedure. Performing pre-procedure clotting blood screening is established and minimizes the risks of developing a spinal hematoma. Early diagnosis of spinal hematoma is required to determine if surgical intervention is required. The IIH Consensus guidelines recommend that serial LPs are not recommended for treatment of headaches in IIH [2].

## Data Availability

Not applicable.

## Abbreviations

LP: Lumbar puncture
CSF: Cerebrospinal fluid
IIH: Idiopathic intracranial hypertension
VP: Ventriculoperitoneal
MRI: Magnetic resonance imaging
CT: Computed tomography
ED: Emergency department
SAH: Subarachnoid hematoma
SDH: Subdural hematoma
IDH: Intradural hematoma
EDH: Epidural hematoma
LMWH: Low molecular weight heparin
AKI: Acute kidney injury
